# Impact of cardiovascular imaging on the management of patients with cardiac masses

**DOI:** 10.1186/1532-429X-18-S1-P133

**Published:** 2016-01-27

**Authors:** Ian C Chang, Dustin Brout, Anne Blaes, Erik B Schelbert, Uma Valeti

**Affiliations:** 1 University of Minnesota, Eagan, MN USA; 2 University of Pittsburgh, Pittsburgh, PA USA

## Background

The anatomical and functional information of a cardiac mass collected by cardiovascular magnetic resonance (CMR) imaging could be clinically important by affecting diagnosis, management and prognosis. Whether CMR impacts clinical care in those with known or suspected cardiac masses is not entirely clear.

## Methods

We identified consecutive patients referred to the University of Minnesota Medical Center ("Minnesota" cohort) between January 1, 2007 and December 31, 2013, and the University of Pittsburgh Medical Center between October1, 2009 and May 30, 2014 ("Pittsburgh" cohort) who were diagnosed with a potential cardiac mass before or after CMR. Final diagnoses were defined by clinician's opinion of histopathology or clinical evidence. Significant clinical impact was defined as CMR establishing a new diagnosis, affecting surgical procedures, or causing medication change as documented in the medical record.

## Results

We identified 34 patients (mean age 56 years, 38% male) with masses in the Minnesota cohort (n = 1621) and 58 patients (mean age 57 years, 48% male) in the Pittsburgh cohort (n = 3358). Overall, CMR resulted in significant impact in 71% (24/34) of the patients in the Minnesota cohort and 72% (42/58) in the Pittsburgh cohort (Table 1). In the Minnesota cohort, CMR established a new diagnosis in 21% (7/34), affected surgical planning in 68% (23/34), and caused medication change in 24% (8/34) of patients. The Pittsburgh cohort yielded similar data.

**Table 1:** Statistics of CMR imaging with significant clinical impact

Category of Clinical Impact	Minnesota Cohort: n (%)	95% CI	Pittsburgh Cohort: n (%)	95% CI
CMR with significant clinical impact	24 (70.6)	0.54-0.83	42 (72.4)	0.60-0.82
Establishing a new diagnosis	7 (20.6)	0.10-0.37	39 (67.2)	0.54-0.78
Affecting invasive or surgical procedures	23 (67.6)	0.51-0.81	23 (39.7)	0.28-0.53
Affecting medication change	8 (23.5)	0.12-0.40	18 (31.0)	0.21-0.44

Values are presented as n (%). CI, confidence interval; CMR, cardiac magnetic resonance imaging.

## Conclusions

CMR of cardiac masses significantly impacts clinical management by facilitating accurate diagnosis and directing clinical management in a substantial percent of patients who had undergone prior evaluations with traditional imaging such as echocardiography. In addition, it also led to avoidance of surgical interventions in masses deemed to be benign or identified as thrombus. CMR is an essential step in the evaluation of majority of patients with cardiac masses or those suspected to be at high risk for developing cardiac masses.

